# Different Scenarios of Autonomous Operation of an Environmental Sensor Node Using a Piezoelectric-Vibration-Based Energy Harvester

**DOI:** 10.3390/s24041338

**Published:** 2024-02-19

**Authors:** Sofiane Bouhedma, Jawad Bin Taufik, Fred Lange, Mohammed Ouali, Hermann Seitz, Dennis Hohlfeld

**Affiliations:** 1 Institute for Electronic Appliances and Circuits, Faculty of Computer Science and Electrical Engineering, University of Rostock, Albert-Einstein-Str. 2, 18059 Rostock, Germanyfred.lange@uni-rostock.de (F.L.); dennis.hohlfeld@uni-rostock.de (D.H.); 2 Structural Mechanics Research Laboratory, Mechanical Engineering Department, Blida I University, BP 270 Route Soumaa-BLIDA, Blida 09000, Algeria; ouali_mohammed@univ-blida.dz; 3 Chair of Microfluidics, Faculty of Mechanical Engineering and Marine Technology, University of Rostock, Justus-von-Liebig-Weg 6, 18059 Rostock, Germany; hermann.seitz@uni-rostock.de

**Keywords:** piezoelectricity, vibration, energy harvesting, microcontroller, sensor node, autonomous operation, Bluetooth low energy

## Abstract

This paper delves into the application of vibration-based energy harvesting to power environmental sensor nodes, a critical component of modern data collection systems. These sensor nodes play a crucial role in structural health monitoring, providing essential data on external conditions that can affect the health and performance of structures. We investigate the feasibility and efficiency of utilizing piezoelectric vibration energy harvesters to sustainably power environmental wireless sensor nodes on the one hand. On the other hand, we exploit different approaches to minimize the sensor node’s power consumption and maximize its efficiency. The investigations consider various sensor node platforms and assess their performance under different voltage levels and broadcast frequencies. The findings reveal that optimized harvester designs enable real-time data broadcasting with short intervals, ranging from 1 to 3 s, expanding the horizons of environmental monitoring, and show that in case the system includes a battery as a backup plan, the battery’s lifetime can be extended up to 9 times. This work underscores the potential of vibration energy harvesting as a viable solution for powering sensor nodes, enhancing their autonomy, and reducing maintenance costs in remote and challenging environments. It opens doors to broader applications of sustainable energy sources in environmental monitoring and data collection systems.

## 1. Introduction

With the continuous advancement of cutting-edge and smart technologies, there is a growing demand for sensing physical phenomena and remote wireless monitoring. Throughout various industrial revolutions, innovative ideas and processes have found their way into industrial applications, leading to the emergence of what we now refer to as “Industry 4.0”. The core concept behind Industry 4.0 is to automate devices and systems, imbuing them with intelligence and connecting them on a global scale, creating what we call Cyber-Physical Systems (CPSs) to enhance human interaction and convenience.

Industrial environments are markedly different from typical everyday work settings, often characterized by harsh conditions that fall outside the realm of optimal human comfort. These conditions include extreme ambient temperatures, varying humidity levels, and the presence of toxic, inflammable, or volatile gases. These extreme factors limit accessibility and can sometimes render these environments hazardous, making access nearly impossible. Hence, the imperative to sense and monitor environmental factors arises, enabling quick response measures and improved interaction with installed devices to enhance work efficiency and automation. One particularly effective approach entails the deployment of wireless sensing systems referred to as “Wireless Sensor Nodes”. These nodes are engineered to operate independently, typically consisting of sensors, a microcontroller, and a radio communication system. To ensure sustained functionality, they are equipped with a battery as their power source, enabling them to operate for extended periods, ranging from several months to a few years, thanks to their remarkable ultra-low-power capabilities.

The substantial growth in ultra-low-power electronics and the subsequent advancements in conditioning circuits have led to a significant reduction in the power consumption of microelectronic systems, including microcontrollers and sensor platforms. This development has spurred the research community to explore the use of energy harvesters, specifically vibration-based energy harvesters, as a viable power source. This category of energy harvesters harnesses ambient energy in the form of vibrations, a common occurrence in industrial environments, to provide power for small electronic devices like sensors, microcontrollers, and wireless transceivers. This breakthrough opens up the potential for the creation of fully autonomous and battery-free systems designed for conventional industrial measurement and monitoring applications. These applications often face limited accessibility due to harsh environmental conditions, making complete battery replacement a formidable challenge. Consequently, the primary objective of our present work is to demonstrate that energy harvesting can supply a sufficient amount of power, thereby extending the lifespan of the currently used battery until the next recharge. Achieving this would significantly reduce waste and the periodic maintenance costs associated with battery replacement.

Numerous research groups have put forth a spectrum of vibration-based energy harvester designs. These designs range from the conventional and simple tip-loaded cantilever-based designs [1,2,3,4] to more intricate and optimized configurations capable of harvesting energy across a broader frequency spectrum [5,6,7,8,9,10,11,12,13,14,15], whereas many other research works have been dedicated to exploring the potential use of different vibration-based energy harvesting to achieve fully autonomous sensing devices designed to be implemented in harsh environments with limited access, and where remote monitoring is needed. Chamanian et al. [16] discussed a practical approach to power wireless sensor nodes using a combination of rechargeable batteries and an electromagnetic vibration energy harvesting system, ensuring reliable and sustainable operation of wireless sensor networks. Zhang et al. [17] introduced a self-powered sensor node that relies on energy harvesting from mechanical vibrations, enabling the autonomous and continuous monitoring of mechanical vibrations, making it particularly valuable for applications where traditional power sources are impractical or costly to maintain. Chamanian et al. [18] studied the possibility of achieving energy-neutral operation in wireless sensor networks (WSNs) through the use of vibration energy harvesting, by developing a system that can sustain itself by harvesting energy from ambient vibrations, ensuring uninterrupted and long-term operation of the wireless sensor network. Lu et al. [19] presented a vibration energy scavenging system designed for micropower applications capable of capturing and utilizing ambient vibration energy to power small electronic devices and sensors. Chamanian et al. [20] presented a novel hybrid energy harvesting system designed to efficiently capture energy from ambient vibrations and capable of powering low-power electronic devices and sensors in remote or inaccessible locations where traditional power sources are unavailable. Elvin et al. [21] performed a feasibility study of vibration-powered sensors for structural monitoring applications and investigated the possibility of harvesting energy using mechanical vibrations present in structures to power sensors used in structural health monitoring systems. Li et al. [22] presented a piezolectric energy harvester designed specifically for capturing energy from ultra-low-frequency vibrations in bridge structures and which can ensure an autonomous structural health monitoring of bridges and different civil infrastructure. Wang et al. [23] presented a comprehensive system-level model of a wireless sensor node powered by piezoelectric vibration energy harvesting, aiming to develop an integrated and efficient system for powering wireless sensor nodes in remote or inaccessible locations. Hadaš et al. [24] presented a model-based approach to designing and testing a vibration energy harvester tailored for aircraft applications, which can enhance the autonomy and efficiency of electronic systems within aircraft. The authors of [25] conducted research on energy conversion from vibrations by employing electromagnetic vibration transducers and explored various design options. Additionally, they demonstrated the feasibility of employing this technology to power wireless sensor nodes. Jang et al. [26] developed a wireless sensor node designed for condition monitoring applications and powered by a vibration energy harvester, which ensures its autonomous operation without relying on conventional power sources. Mouapi et al. [27] presented the design and implementation of an energy harvesting system capable of supplying power to wireless sensor networks (WSNs) deployed in trains, where continuous and autonomous power supply for sensors is crucial for monitoring various aspects of train operations. A novel energy harvesting device, known as the “energy coupler”, has been introduced in [28] to address the power needs of small wireless sensors in power systems. Anisi et al. [29] presented a concise overview of WSN routing and related challenges and provided insights and comparisons between energy harvesting mechanisms and battery-powered routing protocols. The authors of [30] showcased diverse sensor systems that use energy harvesting systems as a power source. The authors of [31] introduced a multimodal hybrid bridge energy harvester that combines piezoelectric and electromagnetic conversion, capable of efficiently transforming vibrations from bridges and harnessing ambient wind energy, to generate useable electrical power to operate wireless sensor nodes (WSNs) for the purpose of health monitoring in bridges. The authors of [32] introduced a self-powered system designed to measure power directly from the tire, subsequently supplying the required energy for a wireless sensor system embedded within the tire. The authors of [33] addressed the topic of energy harvesting, examining its scopes, challenges, and various approaches for use as an alternative energy source and providing a more reliable power supply for wireless sensing systems used in remote and inaccessible areas. In our previous works [34,35,36], we introduced an innovative vibration-based energy harvester design and investigated various methods to enhance the system’s frequency adaptability and broaden its harvesting bandwidth. Furthermore, in a subsequent study [37,38], we presented optimized iterations of our harvester design and conducted experimental demonstrations that showcased enhanced performances.

The development of autonomous and wireless sensor nodes is inherently application-dependent due to their interdisciplinary nature. Consequently, at the time of conducting this research, there was a limited body of work dedicated to the development of autonomous and wireless sensor nodes. The literature on utilizing energy harvesting to power wireless sensor nodes primarily focused on demonstrating the potential of harvested power as a viable energy source. However, these studies often lacked detailed insights into the practical integration of such systems into real-life industrial applications. A notable contribution of our present work lies in conducting a comprehensive investigation and characterization of the entire system, encompassing its final implementation tailored to specific applications and the available power resources. A key novelty is the proposal of a protocol for the practical implementation of the final system. Unlike previous studies, we delve into the intricacies of integrating these systems into real-world industrial scenarios. Furthermore, our research goes beyond showcasing the potential of harvested power, addressing the critical aspect of ensuring the wireless operation of the system. We systematically evaluate the system’s performance under various worst-case scenarios, providing a thorough understanding of its limitations in practical applications. In summary, our paper extends the current literature dealing with autonomous and wireless sensor nodes by offering a detailed exploration of system development, emphasizing real-life industrial applications, and proposing a robust protocol for implementation. Additionally, our study sheds light on the challenges and limitations of employing energy harvesting in ensuring the wireless operation of such systems, enhancing the practical relevance of the research in this domain. We explore various possibilities for establishing an ultra-low-power sensor node designed to capture multiple aspects of the surrounding environment. Specifically, it is tasked with monitoring ambient temperature, air pressure, environmental humidity, and the levels of Total Volatile Organic Compounds (VOCs), which serve as indicators of air quality. The gathered data are wirelessly transmitted to a central host device or server, forming a robust and dependable network. This network serves a crucial role in structural health monitoring, as the aforementioned factors can significantly impact the health and performance of structures.

On the sensing and data acquisition side of the system, we require sensors themselves, either integrated into a single package or distributed across multiple packages. Additionally, an Analog-to-Digital Converter (ADC) is employed to convert these analog data into digital numeric values. A crucial component of this process is a small digital computing processor, typically referred to as a microcontroller, which processes the data into a meaningful format. Our investigation encompasses an evaluation of different microcontroller platforms, as well as an in-depth examination of communication protocols in electronics, including detailed comparisons of their power requirements.

To ensure the system’s reliable operation, even in the event of potential failures, the system integrates a rechargeable Lithium-Ion battery as a backup power source. We have also explored various strategies for receiving transmitted data and decoding embedded information. These strategies aim to minimize power consumption and enable a dependable and autonomous system operation, excluding the battery. Our proposed dual-frequency-vibration-based energy harvester designs play a crucial role in achieving this goal. These harvesters allow us to assess their overall performance under real-world applications. Through an extensive study, we have investigated the influence of broadcast frequencies. The results have successfully demonstrated that the harvesters, when subjected to a harmonic excitation of 0.5 g, can ensure reliable and fully autonomous operation of both the sensing and data transmission system. Notably, in scenarios requiring high broadcast frequencies, such as real-time data reading and transmission, which typically present the most demanding power requirements, our system can extend the battery lifetime by a factor ranging from three to nine times, depending on the microcontroller platform.

## 2. Materials and Methods

### 2.1. The Fundamentals

Before delving into the experimental insights, this section provides an overview of fundamental principles associated with the development of our energy harvesting scheme. This scheme aims to enable the autonomous operation of a sensor platform specifically designed for industrial environments. These principles are intended to facilitate comprehension for readers from various research disciplines, enhancing their understanding of the technical methodologies employed.

#### 2.1.1. Energy Harvesting Module

**Figure 1:**
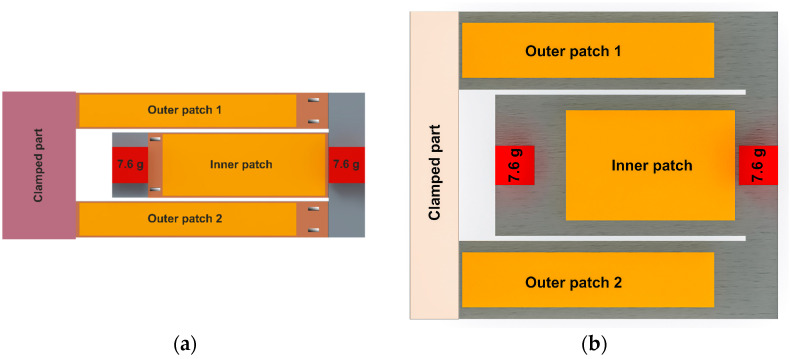
Geometry description of the reference design (**a**) and the optimized design labeled as “Design 1” (**b**) of the vibration-based energy harvester used to power the sensor node [38].

Since the sensor node device is designed to rely exclusively on the output of the vibration-based energy harvester, it is imperative to estimate the available power budget. This estimation will guide our choice of a suitable microcontroller platform, as discussed in [38]. In the latter, we conducted a comprehensive experimental characterization of various energy harvester designs depicted in Figure 1. This characterization allowed us to precisely determine the maximum power output expected from these modules under different levels of harmonic base acceleration.

In Table 1, we provide a summary of the performance metrics for the initial design, referred to as the “reference design”, and its optimized version, consistently labeled as “Design 1” throughout this paper [38]. By using the comparison presented in Table 1, we can extrapolate and estimate the performance of the designs presented in this research work concerning their ability to supply power to a wireless sensing system.

#### 2.1.2. Sensing, Data Acquisition, and Wireless Communication

The primary objective of this work is to investigate different operation scenarios of a low-power sensor node, capable of monitoring the quality of the surrounding environment, using vibration-based energy harvesters. This includes monitoring ambient temperature, air pressure, environmental humidity, and the levels of Volatile Organic Compounds (VOCs), which serve as indicators of air quality. These environmental parameters play a crucial role in determining the suitability of an environment for various activities and also serve as early indicators of potential hazards. The sensing and data acquisition components of the system necessitate the use of sensors, which can be integrated into a single package or distributed across multiple packages. Additionally, an Analog-to-Digital Converter (ADC) is employed to convert the analog data from these sensors into digital numeric values. To process and represent these data in a meaningful format, a small digital computing processor, commonly referred to as a microcontroller, is utilized.

**Figure 2:**
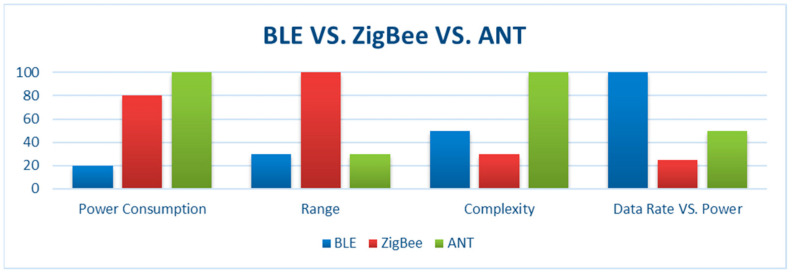
Illustrative comparison between different wireless protocols for IoT based on [39,40,41].

In our pursuit of selecting the most suitable communication protocol for low-power applications, we choose Bluetooth Low Energy (BLE) as the preferred option. This decision is based on several compelling factors, including its widespread availability, ease of implementation, and favorable power-to-performance ratio. A literature survey [39,40,41] has reaffirmed that BLE boasts the lowest power requirements and the highest power efficiency compared to alternative communication protocols, such as ZigBee and ANT, as represented in Figure 2.

Furthermore, BLE has now become an integral component of modern smartphones and laptop computers, which are essential for detecting and processing the broadcast data transmitted by our sensor node. This seamless integration with contemporary consumer devices enhances the practicality and accessibility of our system. On the other hand, Near-Field Communication (NFC) is excluded from our consideration due to its requirement for close proximity, which is not suitable for our application where remote wireless data transmission is a necessity.

In this research, we focus on programming and testing three popular BLE-enabled microcontroller platforms within the ultra-low-power range. One of our objectives is to thoroughly investigate the impact of broadcast frequency on the functionality of the sensing system and data transfer when powered by the energy harvesters discussed in this section. In terms of the communication channel, the transmitting end of our setup comprises a sensor node that includes a microcontroller, sensors, an energy harvesting module, a power management circuit, and optionally, a rechargeable battery. This battery serves as a backup for worst-case scenarios in which the harvesting scheme may fail. On the receiving side, we have the option to use either a smartphone equipped with a suitable BLE application or a computer with the necessary BLE device. We employ a BLE sniffer from “Bluefruit LE Sniffer (BLE 4.0)” (provided by Adafruit Industries^®^, New York, NY, USA), interfaced with a PC and a well-known open source software tool, “Wireshark 4.0” to read the data packets. Our approach involves utilizing a wireless sensor node as a BLE-based non-connectable beacon device operating exclusively in broadcasting mode. To ensure security and data protection, this beacon device does not interfere with other devices; instead, it solely transmits internally stored data. This approach involves intermittent data transmission, with the device returning to a low-power sleep mode in between transmissions. By eliminating receiving capabilities and minimizing transmission time, we aim to achieve the lowest possible power consumption and maximize efficiency.

### 2.2. Overview of the Network

In this section, we aim to provide a comprehensive description of the various elements and attributes within our sensor network. We discuss their operational methodologies and strategies to facilitate seamless intercommunication.

#### 2.2.1. Sensing, Data Acquisition, and Wireless Communication

**Figure 3:**
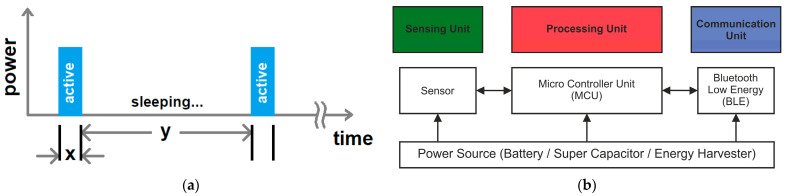
Illustrative presentation of the sensor node broadcast operation (**a**), together with a scheme of internal sub-units of the wireless sensor node (**b**).

Our focus in this work centers on sensing key environmental factors such as ambient temperature, humidity, air pressure, and air quality. Our sensor node is designed to transmit these data wirelessly using the Bluetooth Low Energy (BLE) protocol, which operates within the 2.4 GHz frequency range of the RF spectrum. Given our emphasis on low-power operation, we propose to operate within a DC power supply voltage range of 2.5–3.6 V, which aligns with the standard voltage range for most low-power microcontrollers, including BLE devices and sensors available on the market. The sensor node is configured to spend the majority of its operational life in sleep mode, with active operation occurring in programmed cycles, as illustrated in Figure 3. In each cycle, the system becomes active for a brief period, during which it carries out all necessary activities. It then enters an extended sleep mode until the start of the next cycle, when it wakes up to repeat the process. If the sensor node is active for x seconds and remains in sleep mode for y seconds, we have the following considerations:

- y >> x: this indicates that the sleep time is significantly longer than the active time.
- x + y: this represents the total cycle time (in seconds).
- 1/(x + y): this corresponds to the broadcast frequency (in Hz).

Our low-power scheme under development aims to limit power consumption to below 1 mW, with the aim of continually reducing it to the absolute minimum level achievable. We explore and investigate various strategies and approaches to achieve this objective. Our system employs a single point-to-point network topology, featuring two interconnected devices: a wireless sensor node at one end and a monitoring system at the opposite end. Our sensor node is capable of sensing, processing, and transmitting environmental data wirelessly in brief bursts of data packets. On the other end of the system, a data monitoring device receives the wireless data and displays them. Subsequently, these data can be utilized for various meaningful purposes.

#### 2.2.2. Network Structure

A sensor node is a device designed to detect specific physical attributes in its surrounding environment. It possesses the ability to process these collected data as needed and communicate them to other components within the network. Typically, it includes a portable, long-lasting power source, commonly in the form of a battery housed within its package. In our current setup, we introduce an energy harvesting module connected through a power management board. Figure 3 illustrates the fundamental layout of the proposed sensor node. We propose the utilization of a microcontroller unit equipped with peripheral control modules. These microcontroller processors can be of the 8-, 16-, or 32-bit variety in terms of their operational architecture. Some microcontrollers come with built-in BLE functionalities, while others do not include such capabilities. In cases where the microcontroller lacks integrated BLE functionalities, a separate standalone BLE module needs to be added. Our sensor node also integrates an external ultra-low-power timer module, specifically designed for operations demanding minimal power consumption. Lastly, the sensor node leverages the same power management system and energy storage module as used in [38] for consistency and efficiency.

### 2.3. Hardware and Related Software

In this study, we delve into the possibilities of low-power sensing using BLE as our wireless communication strategy. Our approach involves implementing the sensor node system across three distinct microcontroller platforms. We aim to conduct experimental comparisons of their ultra-low-power performance, ultimately enabling us to identify the most power-efficient microcontroller. Our experimental investigation involves the following microcontrollers:

- The “TI-Launchpad” development board, which is based on the CC2650 microcontroller from Texas Instruments Inc.^®^, Dallas, TX, USA.
- The “Feather-nRF52 Bluefruit-LE” development board, utilizing the Nordic Semiconductor nRF52832 microcontroller and produced by Adafruit Industries^®^, New York, NY, USA.
- A “Custom AVR” development setup, employing the Atmel ATmega328p microcontroller from Microchip Technology Inc.^®^, Chandler, AZ, USA.

The selection of microcontroller platforms is driven by their performance metrics, specifically their capability to operate at the lowest possible voltage levels, ensuring high efficiency. Additionally, ease of access and widespread availability in the market are key considerations in the decision-making process. Through these comparative experiments, we seek to determine the microcontroller platform that offers the best power efficiency for our sensor node system.

The microcontroller is at the center of the sensor node system, and it interacts with various peripheral devices necessary for the functionality of the node. These peripheral modules are essential components that must be interfaced correctly with the MCU, utilizing specific digital interfacing protocols. Specifically, we intend to employ the following modules in conjunction with their respective MCU platforms: The BME680 shuttle board, which interfaces with the MCU to enable environmental sensing. The nRF24L01 wireless module, which facilitates wireless communication and data transmission. The TPL5110 timer breakout board, which plays a critical role in power management and scheduling tasks. By integrating these modules with their respective MCU platforms, we aim to create a fully functional sensor node system capable of efficient low-power sensing and communication. For environmental sensing, we choose the BME680 sensor provided by “Bosch Sensortec GmbH^®^, Reutlingen, Germany”. This sensor is characterized by its ultra-low-power consumption and is capable of measuring environmental parameters such as humidity, pressure, and temperature and the presence of Volatile Organic Compounds (VOCs) or gases. These measurements collectively offer insights into the quality of the surrounding air [42]. Two of the platforms we choose come equipped with built-in BLE features, whereas the Atmega328p does not. Consequently, for the latter, we require an external BLE device to broadcast the collected environmental data. To fulfil this need, we propose utilizing the nRF24L01 2.4 GHz wireless module from “Nordic Semiconductor^®^. Trondheim, Norway”. This module can be programmed to operate as a BLE device, aligning with the objectives of our present work.

To regulate the broadcast frequency of our BLE sensor node, we require an ultra-low-power timekeeping mechanism to wake the MCU up at specified intervals. For the TI-CC2650, an internal operating timer is available even in the lowest power mode, with a power consumption of approximately 50 µA. This offers an alternative option for our operation, which is further investigated in the next section. However, for the nRF52832 and Atmega328p, using an external input as a trigger is the only viable option. Consequently, we rely on the external timer module TPL5110, supplied by Texas Instruments Inc.^®^, Dallas, TX, USA.

In addition to employing BLE as the wireless communication protocol, we also utilize two other standard communication protocols for internal communications between the MCU and peripherals. The BME680 employs the I2C protocol, while the nRF24L01 utilizes SPI for communication with the microcontrollers. At the monitoring station within our sensor network, we have the capability to monitor the data transmitted from the node. This capability serves two primary purposes: allowing the user to retrieve and analyze the environmental conditions and providing the possibility to identify and troubleshoot any potential errors in the development process.

For enhanced practicality, we propose using a smartphone (alternatively a PC) as the monitoring device. Various free apps, designed for different smartphone platforms, are readily available on the market for this purpose. In our research, we primarily utilize a BLE scanner app, which provides comprehensive functionality. It can display essential information about the transmitter device, including its name, attributes such as its MAC address, and any associated data payload in hexadecimal format, as well as raw data. These data packets are user-encodable and -decodable. Additionally, this BLE scanner app offers the capability to assess signal strength, allowing for the estimation of the distance between the receiver and the transmitter.

### 2.4. Power Saving Strategies

A possible power-saving strategy involves utilizing the built-in power-saving schemes integrated into the MCU platform’s software. These schemes are firmware components responsible for activating specific power-saving modes with distinct characteristics [43,44,45], such as sleep mode, deep sleep mode, power-down mode, and power-save mode. They also dictate the conditions under which the MCU should wake up. Typically, these schemes are synchronized with an internal timing mechanism, which allows the MCU system to enter sleep mode for a predefined duration. Wake-up conditions are often triggered by internal or external interrupts.

#### 2.4.1. Reducing the Number of Packets

Reducing the duration of the active broadcast window presents an opportunity to decrease the overall volume of transmitted data packets. This optimization can be achieved through the utilization of BLE APIs, which enable precise control over the timing between individual data packets within a single active broadcast window. By reducing the number of data packets transmitted during each window, we can effectively lower power consumption. Data packet transmissions typically constitute the highest power consumption segments throughout the system’s operation timeline. However, it is important to note that decreasing the number of transmitted packets may pose challenges, particularly in noisy environments. In such cases, receivers may encounter difficulties in reliably detecting the transmitted packets, potentially leading to broadcast failures. Therefore, it is crucial to conduct thorough investigations and establish clear limitations when reducing the number of transmitted packets.

#### 2.4.2. Power Reduction in Hardware

Improving power efficiency can also be attained through hardware optimization and efficient operation. Numerous approaches in this regard have been proposed and extensively examined, as detailed below.

Reducing the Operating Voltage

All microcontrollers (MCUs) and peripheral devices housed within the sensor node function within specific voltage ranges. The system’s efficiency varies across these voltage levels. Our research aims to explore the potential for power consumption reduction by operating the system at the lowest feasible voltage level. Additionally, we conduct an extensive examination of the trade-off between the operating voltage level and overall system efficiency to determine the optimal and most efficient operating configuration.

External Timer Use and I/O Modifications

In this work, we utilize multiple MCU boards, each with distinct capabilities. Notably, the TI-CC2650-Launchpad is the only variant, which includes an active internal timer, enabling it to wake the system up from deep sleep mode. This feature sets it apart from the Adafruit Feather and Atmega328 boards, which lack this internal timer functionality. To compensate for this disparity, we propose incorporating the TPL5110 as an external timer to manage sleep cycles and facilitate wake-up from deep sleep mode. This timer boasts impressive ultra-low-power performance, drawing a mere 35 nA during sleep mode. The research further delves into various configurations employing this timer, providing valuable insights. Additionally, when dealing with the Atmega328p chip, it is advisable to configure the unused I/O to optimize system efficiency.

### 2.5. Autonomous Operation

Analyzing the power consumption curve of the BLE sensor node reveals a very brief active region where the device consumes the most power. Our detailed experimental investigations, outlined in the following sections, highlight that power consumption peaks in these active regions typically require a current supply in the range of 11–15 mA. Providing such high current levels with the proposed energy harvester designs described in previous works [34,35,36,37,38] is an extremely challenging task. Our research has shown that the proposed harvesters can generate power outputs in the milliwatt range, operating at regulated voltages between 1.8 V and 3.6 V, depending on base excitation levels and power management configurations, as summarized in Table 1. Consequently, we suggest considering the incorporation of an energy storage module as a backup plan, capable of supplying the high-current demands characteristic of the active mode. Initially, we explored a sensing system equipped with a coin cell Lithium-Ion battery LIR2032 providing a voltage up to 3.6 V with a 45 mAh capacity as a backup plan. This setup underwent thorough investigation and experimental characterization using various MCUs and at different broadcast frequencies. Our overarching goal is to enable the wireless sensor node to operate autonomously. To achieve this, we intend to explore the possibility of replacing the battery with a suitable supercapacitor providing a voltage up to 5.5 V and a capacity of 470 mF. This supercapacitor would be electrically connected in parallel to the system, thereby contributing to the autonomy of our setup.

### 2.6. Final System Implementation

In this section, we delve into the specifics of the sensor node’s final implementation, which is built upon a microcontroller platform. To keep things simple, we focus on thoroughly explaining the various power-saving strategies integrated into the programming tools associated with the TI-CC2650 Launchpad MCU. It is important to note that further investigations are conducted on all the MCU platforms mentioned earlier. For the final sensor node implementation using the TI-CC2650 Launchpad MCU, we have two viable approaches. One option is to utilize its internal timer, which operates and tracks time even in the ultra-low-power sleep mode. Alternatively, we can opt for an external TPL5110 timer connected to the MCU system.

#### 2.6.1. TI-CC2650 Launchpad MCU + Internal Timer

**Figure 4:**
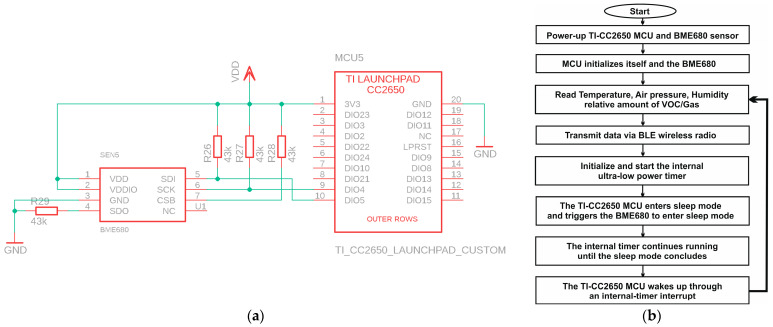
Sensor node electrical circuitry schematic in the case of TI CC2650 MCU + internal timer (**a**), together with its operation flowchart (**b**).

To begin, we propose to connect the BME-680 environmental sensor to the TI-CC2650 Launchpad MCU. The connections between the SCL/SCK and SDA/SDI pins, as well as the DIO4/SCK and DIO5/SDA pins, form the I2C bus. These pins serve as clock and data lines for the I2C connection. To ensure proper operation of the BME-680 sensor, we need pull-up or pull-down resistors on the CSB and SDO pins. Additionally, 43 kΩ resistors are connected to the SDA and SCK pins as pull-up resistors. Furthermore, both the sensor and the MCU share a common power supply. Figure 4 provides a visual overview of the circuit connections.

**Figure 5:**
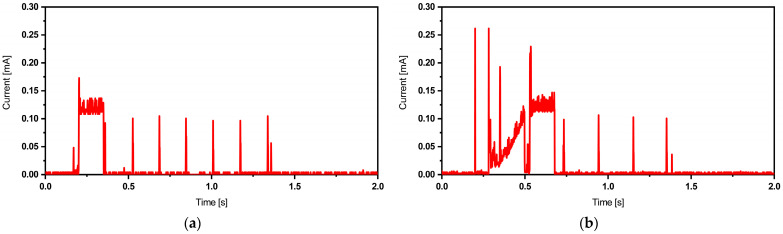
Current traces of BLE packet corresponding to TI-CC2650 Launchpad + internal timer (**a**) and TI-CC2650 Launchpad + TPL5110 timer (**b**).

In this initial scenario, we control the active and sleep times using the MCU’s internal timer. Upon power-up, the MCU initializes itself, and the BME680 sensor begins reading environmental data. After processing and transmitting these data in short bursts via the built-in BLE radio, the MCU puts the sensor to sleep, activates the internal ultra-low-power timer, and enters sleep mode itself. Following a specific sleep duration, the timer generates an internal interrupt, awakening the CPU, and the entire process repeats. Figure 4 outlines the operational flowchart of the device with its internal timer. The BLE transmits data packets in the form of bursts, as depicted in Figure 5, which illustrates the current consumption trace of each BLE. These data are acquired using an oscilloscope via a 10 Ω shunt, with the system operating at a 2.5 V DC voltage. The trace exhibits various activity sections within the packet. Initial spikes correspond to the processor boot-up, followed by a substantial block representing the BME680 sensor activity. Adjacent to this block, there are five prominent spikes indicating the transmission of BLE data packets. A subsequent minor spike indicates MCU processing activity, while smaller spikes during sleep mode are due to internal regulator activities.

Through careful experimental investigation of the TI BLE API, we determine that the lowest achievable broadcast duration is approximately 1 s. Throughout our research, we use an Adafruit BLE sniffer for characterization, which reveals that adopting 5–6 packets per broadcast strikes an optimal balance between low-power consumption and reliable operation. These features are reflected in Figure 5.

#### 2.6.2. TI-CC2650 Launchpad MCU + External Timer

**Figure 6:**
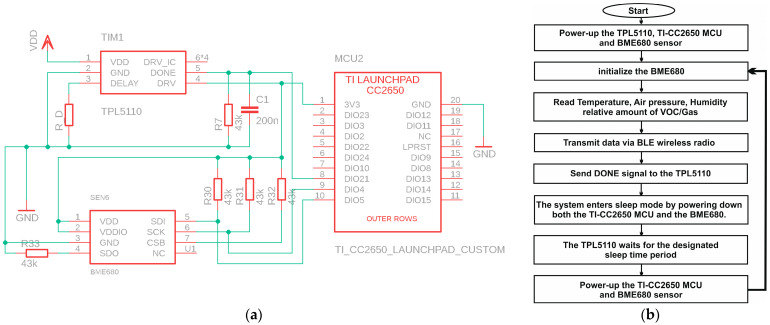
Sensor node electrical circuitry schematic in the case of TI CC2650 MCU + TPL5110 timer (**a**), together with its operation flowchart (**b**).

Another configuration option involves using a TPL5110 timer as an external timer for sleep time control. In this setup, the MCU exclusively determines the broadcast timing. The TPL5110 is an ultra-low-power timer module that requires an external resistor, referred to as “R_DELAY”, to specify the delay time during sleep mode. R_DELAY is connected between the DELAY and GND pins, as outlined in the datasheet [45]. When the entire system starts up, the DRV pin controls the power supply to the load, which includes the MCU and the BME680 sensor in our case, through an onboard MOSFET. While the timer drives a load via the DRV pin, a pulse high at the DONE pin triggers the load to go low and initiates the sleep timing. Typically, this DONE signal is sent to the DONE pin of the TPL5110 timer by the MCU itself. For stable and reliable operation, a pair of resistors and capacitors must be connected in parallel to the DONE pin. Figure 6 provides an overview of the circuitry and electrical connections.

The sleep mode begins when the timer powers down both the MCU and the sensor through the DRV pin. The timer then activates the system by turning on the sensor and MCU at the end of the sleep period. The MCU initializes itself, reads environmental data from the BME680, transmits these data via BLE, and returns to sleep mode. This cycle repeats when a DONE signal, which is a pulse high lasting at least 100 ns, is sent via the DIO21 pin to the DONE pin of the timer. The timer then powers down the system, and the process restarts. The operational flowchart for the TI sensor node circuit with an external timer is illustrated in Figure 6.

Figure 5 displays the BLE packets when the TI-CC2650 Launchpad MCU is used with the TPL5110 external timer module. In comparison to the previous case, differences are observed at the system’s startup phase. The highest power consumption occurs during the timer IC and MCU boot-up. This high-activity phase is followed by the sensor’s current draw, and then the BLE spikes. The sleep mode current, regulated by the timer, measures 35 nA.

Table 2 displays a BLE packet captured in Wireshark, presented as a raw digital data stream. This data stream contains device-specific information, including the device’s MAC address, flags (if any), data length, various fields like company ID and data type, and finally the payload data. The raw hexadecimal data are as follows: “00 07 ba 00 01 86 6d 00 00 d5 0e 00 0c cb 6d 00 00 00 00 00 00 00 00 00”.

## 3. Results

This section focuses on our experimental testing phase. Initially, we aim to assess the power consumption of various sensor nodes across three MCU platforms. This evaluation encompasses different broadcast frequencies and voltage levels to pinpoint the most power-efficient operating range. Following that, we intend to explore the feasibility of running the system with the energy harvesters. To ensure comprehensiveness, we conduct a thorough experimental investigation involving different operating scenarios. These scenarios include using a Lithium-Ion battery as an energy storage module with a contingency plan for system failures. Additionally, we explore the possibility of operating the system entirely autonomously, considering a supercapacitor as an alternative energy storage module capable of providing the high boot-up current required for reliable system operation. Furthermore, we experimentally assess the extension of battery life achievable by incorporating the proposed harvesters when a battery serves as a backup system.

### 3.1. Power Consumption Measurement Strategy

**Figure 7:**
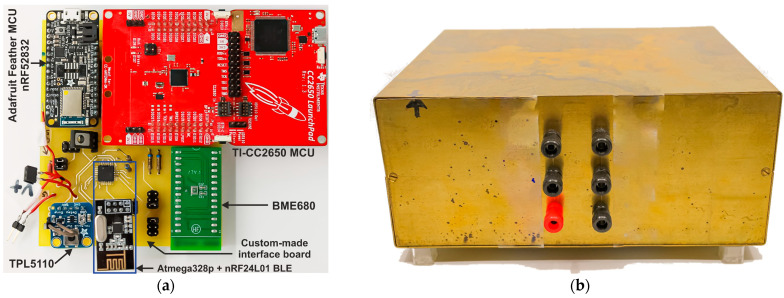
Overview of the test board used to connect different MCU platforms where only the tested MCU is connected during the characterization (**a**), and the designed shielding box used during the experimental test to omit RF disturbance from external environment (**b**).

We have established an experimental test bench comprising a Keysight^®^ MSOX3054A Mixed Signal oscilloscope, a Rigol^®^ DM3058E multimeter, and a GW Instek^®^ GPS4303 power supply unit (PSU). This setup enables us to precisely measure the power consumption of the three sensor nodes. We provide the sensor nodes with a constant DC voltage supply within the recommended range of 2–3.5 V from the PSU. To calculate power consumption accurately, we monitor the RMS value of the variable DC current that characterizes the sensor node’s activity. Our multimeter boasts five-and-a-half digits of resolution, making it capable of measuring currents in the nA range. Additionally, we employ the oscilloscope to visually analyze the current signal during broadcasting. To ensure precision and repeatability, we run the system for an extended period, collecting a sufficient number of samples for accurate measurements. Given the ultra-low-power nature of our electronics, and for enhanced accuracy and repeatability, we propose conducting these tests inside a shielded brass-based enclosure, as shown in Figure 7. This enclosure helps eliminate the influence of external RF disturbances on our measurements.

Our systems exhibit a current consumption profile comprising both AC and DC components. To accurately capture this, we employ a two-fold approach for each measurement. First, we record readings in AC mode to isolate and exclude all DC components. Then, conversely, we record readings in DC mode. By applying the formulas detailed in Equation (1), we can calculate the total I_RMS_ value, representing the combined electrical current consumption of the system. This approach allows us to accurately account for both AC and DC components in our measurements [46]:

(1) $$I_{RMS}=\sqrt{I_{AC}^{2}+I_{DC}^{2}}.$$

To address potential inaccuracies arising from the variable duration of our nodes’ cyclic operation, ranging from 5 s to 10 min, we have divided the measurement into two procedures. The first part focuses on the active region, while the second part concentrates on the sleep mode. These two segments are then combined to yield final calculations. Given that each of our sensing platforms maintains an active broadcast duration of around 2 s, we programmed each node to perform real-time readings, equating to one broadcast every 2 s (1 Broadcast/2 s). We acquired RMS current values over a two-minute interval, encompassing 60 broadcast packets. Averaging these RMS current samples allows us to determine the actual steady-state current consumption during the active phase. We then executed the same measurement procedure to ascertain the sleep current. By applying the formula in Equation (2), we can subsequently compute the system’s power consumption. This approach helps us address variations in operation duration and ensures accurate power consumption calculations [46].

(2) $$P_{RMS}=\frac{\left(2\times I_{RMS}^{active}\;\right)+\left[I_{RMS}^{sleep}\times \left(t_{cycle}-2\right)\right]}{t_{cycle}}\times V_{DC\;},$$

where $P_{RMS}$ is the total RMS power consumption, $I_{RMS}^{active}$ is the RMS current value measured in the active broadcast region, $I_{RMS}^{sleep}$ is the RMS current value measured during the inactive sleep region, $t_{cycle}$ is the total broadcast time interval or system cycle time, and $V_{DC}$ is the DC voltage supply.

Table 3 provides a summary of power consumption data for our sensor nodes, utilizing different MCU platforms. These measurements were conducted at 2.5 V and 3.0 V DC voltage supplies, and various broadcast intervals of 5, 15, 30, 60, 120, 300, and 600 s. Additionally, we determined the equivalent resistances exhibited by the nodes at different power consumptions. Our experiments have revealed that the sensor node integrated with the ATMEGA328P MCU stands out as the most power-efficient platform among the options. However, we encountered reliability issues when operating the system with a 2.5 V voltage supply.

**Figure 8:**
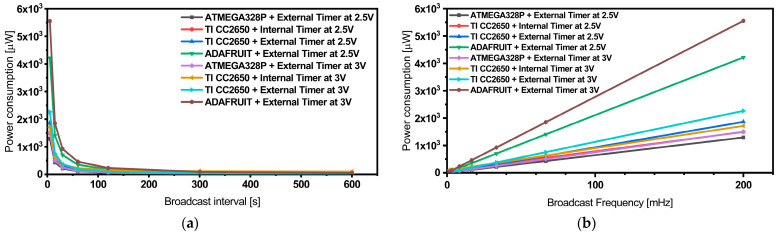
Quantitative power consumption comparison of the different sensor nodes measured using a DC power supply vs. broadcast interval (**a**) and broadcast frequency (**b**).

It became evident that this MCU platform requires a minimum voltage supply of 2.7 V for reliable operation. Another strong contender is the TI-CC2650 Launchpad, which showcases comparable power consumption levels and consistently reliable operation. Our data also suggest that employing the TI-CC2650 Launchpad MCU with the internal timer can be advantageous for reducing power consumption, especially in applications with broadcast time intervals longer than 30 s. However, for shorter broadcast intervals, the power consumption tends to be comparable or even slightly higher, as illustrated in Figure 8.

As the next step, we explore the viability of using our proposed harvester designs, depicted in Figure 1, as sustainable power sources for our sensing system. We utilized the same power management and conditioning circuits as described in [38]. This investigation comprises two main parts. Initially, we examined the system’s performance with a Lithium-Ion battery serving as a power storage unit and a backup plan for potential system failures. Subsequently, we replaced the battery with a supercapacitor to deliver the high current required during the active phase. This allowed us to assess the possibility of operating the entire system autonomously without the need for a traditional battery.

### 3.2. Sensor Node Operation with an Energy Harvesting Scheme as a Power Supply and a Battery as a Backup

In the first scenario, our system utilizes the dual-frequency-vibration-based energy harvester’s reference design, which was comprehensively characterized in [38]. This harvester is employed to power our sensor node, integrated with the same conditioning circuitry and power management scheme (DC2151A provided by Analog Devices Inc.^®^, Wilmington, MA, USA) used for the harvester’s characterization. The power management board can connect a 3.6 V coin-cell Lithium-Ion battery, serving both as a storage unit and a backup power supply. This board, equipped with internal circuitry and a prioritizer, allows seamless switching between different power sources, in our case, the energy harvester and the Lithium-Ion battery. However, as shown in Table 1, the dual-frequency-vibration-based energy harvester alone cannot supply the high current levels required to initiate the system. To overcome this limitation, we used the battery to provide the necessary high current spikes during the active mode while storing harvested energy during the sleep mode phase in the battery.

**Figure 9:**
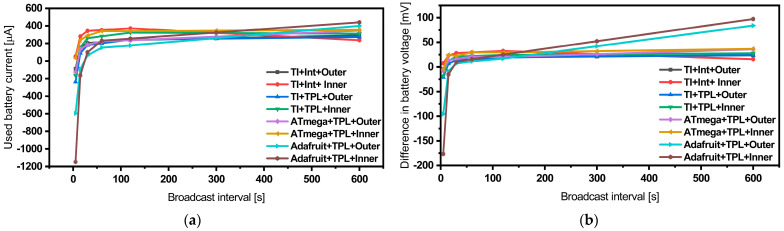
Battery’s used current level (**a**) and its equivalent voltage difference (**b**) experimentally measured at different broadcast time intervals while operating the different sensor nodes using the energy harvester (outer and inner patches) as a main power supply and a battery as a backup for the worst-case scenarios.

To conduct these tests, we used the same test bench and vibration equipment described in [36,37,38]. During the experimental characterization, we subjected the energy harvester to harmonic base-acceleration with a value of a = 0.5 g. We also tested the entire sensing system, incorporating the three MCU platforms, at various broadcast frequencies and input voltage levels. For the sake of simplification, we assumed a 65% charge level for the LIR2032 coin-cell battery, providing a nominal voltage of 3.6 V and a capacity of 45 mAh, which operates within the linear regime. The system characterization involved monitoring the battery’s voltage and current levels over a duration of 30 min. Negative current values indicate discharge behavior, signifying that despite the harvester’s presence, the system will eventually fail after a certain period of time. Detailed long-term experimental test data are shown in Figure 9 and Table 4.

**Figure 10:**
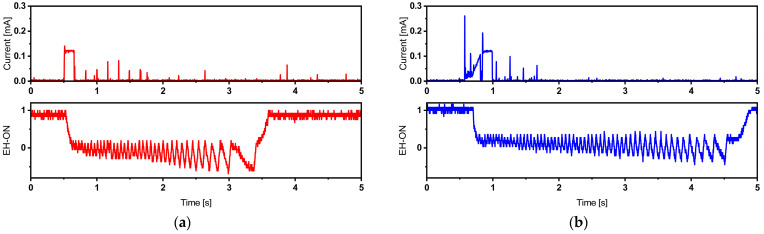
Current traces of BLE packet corresponding to TI-CC2650 Launchpad + internal timer operating at 5 s broadcast time interval (**a**) and TI-CC2650 Launchpad + TPL5110 timer operating at 7 s broadcast time interval (**b**), illustrating the EH-ON signal read directly from the power management source DC2151-A and indicating the used power source. Different colors are employed to differentiate between the two operating configurations.

The power management board includes an “EH-ON” pin that generates a logical signal. This signal is in a high state when the system is powered by the harvesting source, and it transitions to a low state when the system draws power from the battery. Figure 10 provides a visual representation of the current consumption signal during a single broadcast, overlaid with the EH-ON signal. This illustrates that the system primarily relies on the battery to deliver the high current spikes required during the active broadcast phase.

The presence of a harvesting source prompts the system to swiftly recover and switch back to utilizing the harvesting source for power. This behavior is evident in the EH-ON signal, which intermittently flickers but does not consistently drop to a low state during the active region’s power demands.

**Figure 11:**
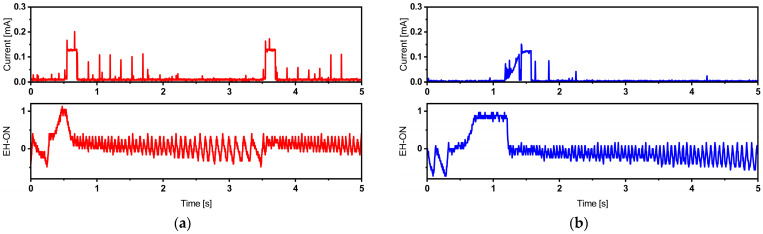
TI-CC2650 autonomous operation failure, indicated by the non-recovering EH-ON signal measured on the DC2151-A power management board, corresponding to TI-CC2650 Launchpad + internal timer (**a**) and TI-CC2650 Launchpad + TPL5110 timer (**b**). Different colors are employed to differentiate between the two operating configurations.

As described in Table 4, when using a short broadcast time interval (5 s for the TI-CC2650 Launchpad and ATmega328p MCUs, and 15 s for the Adafruit Feather), negative battery current values are observed. This indicates that the backup battery is discharging. Over time, this continuous discharge will deplete the battery, eventually resulting in a system failure. Figure 11 provides a visual representation of the EH-ON signal recorded in such cases.

In this scenario, it is apparent that the system struggles to recover and switch back to the energy harvesting source. Consequently, the EH-ON signal continuously flickers around the Low value. In such cases, the harvester serves as a battery life extender, meaning that it prolongs the time it takes for the battery to fully discharge, delaying the need for replacement. To empirically determine the battery life extension coefficient, we propose conducting a specific test. First, we operate the system for 30 min at a 5 s broadcast time interval, exclusively relying on the battery (a “battery-only” mode) while excluding the energy harvesting source. Subsequently, we switch the system to a “battery + energy harvester” mode. By utilizing the capacity of the Lithium-Ion battery and the recorded discharge current, we can estimate the time required for the battery to become fully discharged. This estimation is performed in accordance with equations such as Equations (3)–(5) [47,48].

(3) $$C_{battery}=I_{battery}\times t$$

(4) $$\begin{array}{c} t_{discharge}^{battery-only}=\frac{C_{battery}}{I_{discharge}^{battery-only}},\\ t_{discharge}^{battery+harvestr}=\frac{C_{battery}}{I_{discharge}^{battery+harvester}}, \end{array}$$

(5) $$r=\frac{t_{discharge}^{battery+harvester}}{t_{discharge}^{battery-only}}\;$$

where $C_{battery}$ is the nominal battery capacity, $I_{battery}$ is the battery current, t is the time, $t_{discharge}^{battery-only}$ is the battery discharge time in the battery-only mode, $I_{discharge}^{battery-only}$ is the recorded discharge current in the battery-only mode, $t_{discharge}^{battery+harvestr}$ is the battery discharge time in the battery+harvester mode, $I_{discharge}^{battery+harvester}$ is the recorded discharge current in the battery + harvester mode, and r is the battery-life-extension coefficient. Table 5 summarizes the battery-life-extension coefficient for the different sensor nodes.

To validate our estimation of battery life extension, we compared our estimated results with direct measurements using the MAX17055XEVKIT Evaluation Kit provided by Maxim Integrated^®^, San Jose, CA, USA. The data presented in Table 5 clearly demonstrate the benefits of integrating our energy harvester as a power supply for our system, even in the worst-case scenarios where the system relies solely on the battery. In such situations, the harvester effectively extends the battery life. Our results show that this approach can increase battery life by a factor ranging from 2.8 up to approximately 9 times. This significant life extension feature has a substantial impact, notably reducing the frequency of battery replacements and associated maintenance costs.

### 3.3. Autonomous Operation of Sensor Nodes with PVEH

Building on our previous results, which demonstrated the feasibility of operating our system with the energy harvester alongside a backup battery for challenging scenarios, we were inspired to explore the potential for fully autonomous operation. To achieve this, we suggest removing the Lithium-Ion battery and substituting it with a supercapacitor. This supercapacitor will be electrically connected in parallel to the system and will play a crucial role in supporting the high switching impulses and radio transmission peaks in our BLE-based node circuits. The power management board already includes a 20 mF supercapacitor connected to the output. However, this capacity has proven insufficient to power the node systems effectively. As a solution, we recommend adding an additional commercially available 470 mF supercapacitor, which provides a nominal voltage of 5.5 V (the same as the capacitor used in [38]). Experimental tests have shown that this chosen capacitance represents the minimum threshold required to reliably operate the system at various broadcast frequencies. Any capacitance lower than this value results in a failure to power the sensor node adequately.

**Figure 12:**
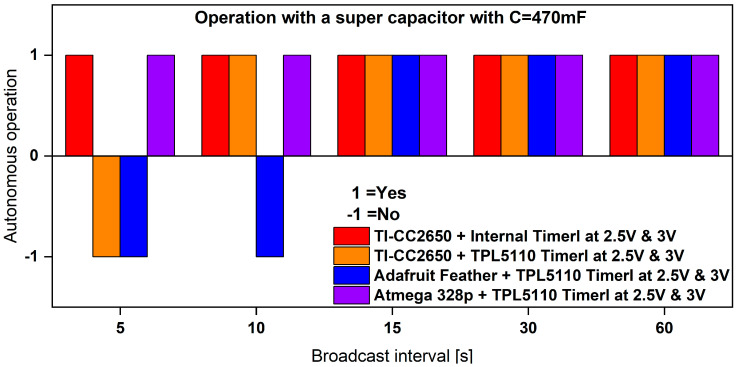
Summary of supercapacitor-backed autonomous operation possibilities of sensor nodes with our proposed energy harvester.

We conducted experimental tests under identical conditions as those for the battery, with a duration of 10 min. The investigation encompassed the same broadcast time intervals, as illustrated in Figure 12. Building upon insights gained from previous investigations, we determined the maximum broadcast frequency for each sensor node, serving as a performance threshold. Consequently, this threshold served as the starting point for our current investigations. Our primary objective was to ascertain the minimum broadcast time interval (equivalent to the highest broadcast frequency) required for autonomous operation of the sensor nodes. To accomplish this, we leveraged the characteristics of the supercapacitor and introduced Equation 6 for our analysis:

(6) $$dI=C\times \frac{dV}{dt},$$

where dI is the current variation flowing into the supercapacitor, C is the capacitance, dV is the supercapacitor variation, and finally dt is time in seconds.

In an ideal scenario, the current consumption of a sensor node should be at 0 mA. However, in practical situations, there is always a minimal current flowing through the capacitor. Throughout our tests, we observed a consistently stable voltage across the capacitor/load. You can find a summary of the experimental test data in Table 6.

These results reaffirm our previous findings, indicating that the most power-efficient sensor nodes are those incorporating the ATMEGA328P and TI-CC2650 MCUs. We observed that the ATMEGA328P MCU requires a voltage supply higher than 2.7 V for reliable operation, so the corresponding tests were conducted at a 3 V voltage level. In contrast, the TI-CC2650 operates reliably at a minimum voltage level of 2 V. However, for practicality and comparability, we conducted the experimental characterization with a voltage supply of 2.5 V. The lower operational voltage level is a significant feature for certain real-life applications that demand a minimal voltage supply. To explore this further, we exclusively tested the sensor node incorporating the TI-CC2650 MCU alongside an updated vibration-based energy harvester known as “Design I”, capable of providing higher power levels. Table 7 provides an overview of the harvester’s performance as a power supply for our sensor node.

These results highlight that the use of the optimized harvester design creates the opportunity to broadcast and monitor environmental data in real-time, particularly in scenarios requiring broadcast intervals as short as 1–3 s.

## 4. Conclusions and Outlooks

In this work, we programmed three microcontroller platforms and successfully developed Bluetooth Low Energy (BLE)-based wireless sensor nodes capable of sensing and transmitting environmental data such as temperature, air pressure, humidity, and air quality over a standard protocol distance of 30 m. The overarching goal of achieving autonomous operation in an ultra-low-power mode through the implementation of a vibration-based energy harvesting scheme has been realized.

Various hardware and software power-saving techniques have been implemented and thoroughly studied to minimize the power consumption of our system. This was achieved through the optimization of timing protocols, implementation of multiple power-saving schemes, utilization of different sleep modes, tweaking operating voltage levels, and integrating ultra-low-power external timing devices where necessary.

Furthermore, we conducted a comprehensive investigation of worst-case system operation scenarios, where a battery serves as a backup power supply. Our results demonstrated that by employing our energy harvesting source, the battery lifespan can be extended by a factor of 9 times. This feature holds significant potential for reducing maintenance costs and minimizing battery waste.

To enhance the system’s reliability, we implemented various measures aimed at improving robustness and noise tolerance within the feedback system. As part of future improvements, we propose upgrading the system by substituting the existing external timer with a more dependable timer module.

Finally, throughout this work, a 2.45 MHz MCU speed clock was used for all platforms. Exploring the possibility of lowering this value could be another avenue for optimizing the ultra-low-power operation of the sensor node. While potential reliability issues in operation might arise, it is worth studying the tradeoff between a reliable operation, a slower MCU speed clock, and power consumption. Furthermore, the system integration of the overall sensing unit can be enhanced, especially in scenarios with space restrictions. This can be achieved by the development of a dedicated circuit board, which would consolidate the microcontroller, the power management board, and the sensing unit’s Integrated Circuits onto a single platform. This approach would result in optimized space utilization by omitting all non-used units, contributing to a more streamlined and efficient design. In addition, real-life tests under realistic industrial environmental conditions are planned, utilizing ambient vibrations generated by ship engines and various rotating machines. These tests aim to provide a deeper understanding of the operational capabilities of our sensing system. However, a redesign of the harvesters and an extension of the current system specifications will be necessary to ensure effective operation in such challenging and harsh environments.

## Figures and Tables

**Table 1:** Overview of the available generated power from the energy harvesters used in this research, under a 0.5 g base-acceleration according to [38].

	Reference Design		Design 1 (Optimized)	
	Mode 1	Mode 2	Mode 1	Mode 2
Base acceleration [g]	0.5			
Frequency [Hz]	65.03	77.78	72.12	76.85
Voltage output (2151A) [V]	2.5			
Current output [mA]	0.638	1.074	3.398	1.537
Power output [mW]	1.595	2.685	8.495	3.842

**Table 2:** Interpretation of the payload raw data received in hexadecimal format.

N° of Bytes	Hexadecimal	Decimal	Parameter	Reading	Unit
3	00 07 ba	1978	Temperature	19.78	[°C]
4	00 01 86 6d	99949	Pressure	99949	[Pa]
4	00 00 d5 0e	54542	Relative humidity	54.542	[%]
4	00 0c cb 6d	838509	Air quality	838509	[Ω] ^(1)^

^(1)^ Very high resistance indicates a high air quality.

**Table 3:** Experimentally measured power consumption of each sensor node using different MCU platforms at all possible configurations and at different broadcast frequencies.

MCU Platform	DC Supply [V]	Broadcast Time Interval [s]						
		5	15	30	60	120	300	600
		Power Consumption [µW]						
ATMEGA328P + TPL5110 TIMER	**2.5**	1291.4	431.79	216.88	109.43	55.700	23.464	12.719
TI-CC2650 + INTERNAL TIMER		1493.6	550.01	314.09	196.14	137.16	101.78	89.980
TI-CC2650 + TPL5110 TIMER		1863.6	621.28	310.68	155.39	77.740	31.151	15.621
ADAFRUIT + TPL5110 TIMER		4217.5	1405.9	702.99	351.54	175.81	70.374	35.228
ATMEGA328P + TPL5110 TIMER	**3**	1502.5	502.71	252.77	127.80	65.318	27.827	15.329
TI-CC2650 + INTERNAL TIMER		1713.9	631.40	360.75	225.43	157.77	117.17	103.64
TI-CC2650 + TPL5110 TIMER		2263.8	754.68	377.40	188.75	94.433	37.840	18.976
ADAFRUIT + TPL5110 TIMER		5553.1	1851.1	925.61	462.86	231.49	92.662	46.387

**Table 4:** Detailed overview showing the performances of the sensor node, which uses different MCU platforms at different broadcast frequencies using the reference energy harvester’s power generated at each mode, backed up with a Lithium-Ion battery for the worst-case scenarios.

Configuration	Broadcast Interval [s]	Battery Readings		Configuration	Battery Readings	
		I [µA]	∆V [mV]		I [µA]	∆V [mV]
**TI-CC2650 + Internal Timer** Vibration mode: Mode I Frequency: 65.03 Hz Acceleration: 0.5 g	5	−88.545	−4.54	**TI-CC2650 + Internal Timer** Vibration mode: Mode II Frequency: 77.78 Hz Acceleration: 0.5 g	54.464	7.59
	15	152.693	12.52		280.695	23.03
	30	205.009	20.98		343.178	28.26
	60	216.362	20.789		352.485	29.683
	600	292.24	23.613		236.76	15.783
**TI-CC2650 + TPL5110 Timer** Vibration mode: Mode I Frequency: 65.03 Hz Acceleration: 0.5 g	5	−236.792	−20.8	**TI-CC2650 + TPL5110 Timer** Vibration mode: Mode II Frequency: 77.78 Hz Acceleration: 0.5 g	−164.394	−18.08
	15	90.608	6.52		154.443	12.43
	30	180.759	13.92		260.329	21.12
	60	201.763	15.753		281.232	22.206
	600	273.542	23.98		311.848	27.82
**ATmega328p + TPL5110 Timer** Vibration mode: Mode I Frequency: 65.03 Hz Acceleration: 0.5 g	5	−125	−7.85	**ATmega328p + TPL5110 Timer** Vibration mode: Mode II Frequency: 77.78 Hz Acceleration: 0.5 g	35.421	−4.307
	15	134.347	13.934		237.984	24.69
	30	185.271	16.045		288.483	22.36
	60	224.398	20.82		340.553	29.53
	600	346.33	35.962		354.36	36.703
**Adafruit Feather + TPL5110 Timer** Vibration mode: Mode I Frequency: 65.03 Hz Acceleration: 0.5 g	5	−595.385	−95.428	**Adafruit Feather + TPL5110 Timer** Vibration mode: Mode II Frequency: 77.78 Hz Acceleration: 0.5 g	−1147.73	−176.6
	15	−91.645	−8.488		−162.34	−15.04
	30	64.184	7.047		99.94	9.82
	60	152.909	11.351		232.06	16.1
	600	400.08	84.002		440.46	97.258

**Table 5:** Experimental results of the expected battery life extension when operating the sensor node using the energy harvester and the battery as a backup plan in the worst-case scenarios.

Configuration		Operation Mode				Results
		Battery + Harvester		Battery-Only		
MCU Platform	Broad. Interval [s]	I [µA]	∆V [mV]	I [µA]	∆V [mV]	r
TI-CC2650 + TPL5110 Timer	5 s	−164.394	−18.08	−457.711	−0.37	2.78
ATmega328p + TPL5110 Timer	5 s	−35.42	−4.307	−340.422	−25.68	9.61
Adafruit Feather + TPL5110 Timer	15 s	−162.34	−15.04	−455.73	−3.86	2.81

**Table 6:** Overview showing the autonomous operation of the sensor node, which uses different MCU platforms at different broadcast frequencies using the reference energy harvester’s power generated at each mode stored in a supercapacitor.

Configuration	E.H.	*V_output_* [V]	Br. Time Interval [s]	*I_capacitor_* [µA]	*V_capacitor_* [V]
ATMEGA328P + TPL5110 TIMER	**Reference Design**	**2.5**	5	n/a	n/a
TI-CC2650 + INTERNAL TIMER			5	3.372	2.477
TI-CC2650 + TPL5110 TIMER			6	0.877	2.476
ADAFRUIT + TPL5110 TIMER			15	1.467	2.475
ATMEGA328P + TPL5110 TIMER		**3**	5	18.801	2.972
TI-CC2650 + INTERNAL TIMER			5	11.598	2.972
TI-CC2650 + TPL5110 TIMER			6	10.717	2.971
ADAFRUIT + TPL5110 TIMER			15	3.091	2.968

**Table 7:** Performances of the autonomous sensor node operating using the optimized energy harvester (labeled as “Design 1”), providing higher power levels compared to the reference design.

Configuration	E.H.	Mode	*V_output_* [V]	Br. Time Interval [s]	*I_capacitor_* [mA]	*V_capacitor_* [V]
TI-CC2650 + INTERNAL TIMER	**Design I**	I	**2.5**	1	3.62	2.49
		II		3	1.12	2.48
TI-CC2650 + TPL5110 TIMER		I		1	3.44	2.48
		II		3	2.08	2.49

## Abbreviation

**Abbreviation**	**Full Form**	**Abbreviation**	**Full Form**
AC	Alternating Current	MCU	Microcontroller Unit
AD	Analog Devices	MISO	Master In Slave Out
ADC	Analog-to-Digital Converter	MOSFET	Metal–Oxide–Semiconductor Field Effect Transistor
PVEH	Piezoelectric-Vibration-based Energy Harvester	PC	Personal Computer
ANT	Adaptive Network Topology	PDU	Protocol Data Unit
API	Application Programming Interface	PM	Power Management
AVR	Alf and Vegard’s RISC processor	PSU	Power Supply Unit
BLE	Bluetooth Low Energy	RF	Radio Frequency
CPS	Cyber-Physical Systems	RISC	Reduced Instruction Set Computing
CPU	Central Processing Unit	RMS	Root Mean Square
DC	Direct Current	RTOS	Real-Time Operating System
DRV	Driving point of load	SCL	Serial Clock in I2C
GND	Ground	SCLK	Serial Clock in SPI
IC	Integrated Circuit	SDA	Serial Data in I2C
ID	Identification	SDK	Software Development Kit
IDE	Integrated Development Environment	SM	Security Manager
IoT	Internet of Things	SPI	Serial Peripheral Interface
I2C	Inter-Integrated Circuit	TI	Texas Instruments
I/O	Input/Output	USB	Universal Serial Bus
Li-ion	Lithium Ion	VDD	Voltage Drain Supply (DC Supply point of a circuit)
MAC	Medium-Access Control	VOC	Volatile Organic Compound
